# Rational design of unrestricted pRN1 derivatives and their application in the construction of a dual plasmid vector system for *Saccharolobus islandicus*

**DOI:** 10.1002/mlf2.12107

**Published:** 2024-03-20

**Authors:** Pengpeng Zhao, Xiaonan Bi, Xiaoning Wang, Xu Feng, Yulong Shen, Guanhua Yuan, Qunxin She

**Affiliations:** ^1^ CRISPR and Archaea Biology Research Center, State Key Laboratory of Microbial Technology and Microbial Technology Institute Shandong University Qingdao China

**Keywords:** archaeal genetics, CRISPR‐Cas, CRISPR escape mutations, dual plasmid vectors, *Saccharolobus–E. coli* shuttle vectors

## Abstract

*Saccharolobus islandicus* REY15A represents one of the very few archaeal models with versatile genetic tools, which include efficient genome editing, gene silencing, and robust protein expression systems. However, plasmid vectors constructed for this crenarchaeon thus far are based solely on the pRN2 cryptic plasmid. Although this plasmid coexists with pRN1 in its original host, early attempts to test pRN1‐based vectors consistently failed to yield any stable host–vector system for *Sa. islandicus*. We hypothesized that this failure could be due to the occurrence of CRISPR immunity against pRN1 in this archaeon. We identified a putative target sequence in *orf904* encoding a putative replicase on pRN1 (target N1). Mutated targets (N1a, N1b, and N1c) were then designed and tested for their capability to escape the host CRISPR immunity by using a plasmid interference assay. The results revealed that the original target triggered CRISPR immunity in this archaeon, whereas all three mutated targets did not, indicating that all the designed target mutations evaded host immunity. These mutated targets were then incorporated into *orf904* individually, yielding corresponding mutated pRN1 backbones with which shuttle plasmids were constructed (pN1aSD, pN1bSD, and pN1cSD). *Sa. islandicus* transformation revealed that pN1aSD and pN1bSD were functional shuttle vectors, but pN1cSD lost the capability for replication. These results indicate that the missense mutations in the conserved helicase domain in pN1c inactivated the replicase. We further showed that pRN1‐based and pRN2‐based vectors were stably maintained in the archaeal cells either alone or in combination, and this yielded a dual plasmid system for genetic study with this important archaeal model.

## Impact statement

When pRN1 was employed for vector construction in *Saccharolobus islandicus* REY15A, pRN1‐derived vectors were not stable in this archaeon. Here, we show that pRN1 *orf904* encoding a putative replicase on pRN1 carries a DNA segment to be targeted by the host I‐A CRISPR system. By designing mutated target sequences that evade the CRISPR immunity, efficient plasmid vectors were obtained with mutated pRN1 backbones. This strategy could be applied in developing host–vector systems for other microorganisms with plasmids or viruses carrying CRISPR target sequences. Moreover, the resulting dual vector system would facilitate genetic studies with this important crenarchaeal model.

## INTRODUCTION

Archaea were first discovered as the third domain of life by Carl Woese and colleagues in the 1970s in their pioneer phylogenetic study of microorganisms based on small subunit rRNA sequences^1^, ^2^, ^3^. This classification was then confirmed by subsequent genomic studies of several archaea and bacteria, since genome sequence analysis revealed that archaea share information‐processing machineries with Eukarya, such as proteins/enzymes responsible for DNA replication and RNA transcription^4^. Archaea also exhibit unique cell biology^5^. Over 20,000 archaea are known, comprising 30 phyla^6^. In particular, the discovery of archaea belonging to the Asgard superphylum represents one of the most exciting research breakthroughs in the contemporary biology. These archaea are probably ancestors of eukaryotes, since they share hundreds of proteins involved in cellular processes such as membrane trafficking with eukaryotes^7^, ^8^, ^9^. Therefore, archaeal models are important not only for the investigation of novel biological principles in their own rights but also for exploring the origin of eukaryotes and the evolution of eukaryotic biological features^5^, ^10^, ^11^.

Currently, archaeal genetic and functional studies are still limited to a few model organisms belonging to the two classical phyla, *Euryarchaeota* and *Crenarchaeota* ^9^, ^12^, ^13^, ^14^, among which *Sulfolobus acidocaldarius* and *Saccharolobus islandicus* of the latter phylum represent the genetically tractable archaeal models^15^, ^16^, ^17^ that are more closely related to Asgard archaea, the predicted most recent ancestor of eukaryotes. Since their discovery by metagenomic analysis of marine samples in 2015^18^, great cultivation efforts have yielded only two Asgard cultures, and both organisms grow extremely slowly^19^, ^20^, a feature that precludes them from being developed as genetic models in the near future. Consequently, other models need to be employed for the genetic and functional characterization of the eukaryotic signature proteins identified in Asgard archaea. In this regard, *Sulfolobales* organisms, such as *S. acidocaldarius* and *Sa. islandicus*, serve as important models for these investigations.

Versatile genetic tools are available for *S. acidocaldarius* and *Sa. islandicus* ^15^, ^16^, ^17^, ^21^. However, vectors constructed for the former are based on pRN1^22^, whereas those constructed for the latter are based on pRN2^23^. Interestingly, both cryptic plasmids coexist in *Sa. islandicus* REN1H1, the original host^24^, suggesting that they could provide compatible archaeal origins of replication for developing a dual vector system in *Sa. islandicus*.

*Sa. islandicus* REY15A was isolated from an enrichment culture generated from hot spring samples collected in the Reykjavík region of Iceland^25^, and it grows optimally at 78°C, pH 3.5^26^. This archaeon has been a model for the investigation of antiviral mechanisms by different CRISPR–Cas systems, including I‐A and III‐B subtypes that employ ribonucleoprotein complexes of multiple subunits to mediate nucleic acid interference in a small RNA‐guided fashion^27^, ^28^, ^29^. In archaea biology research, studies with this crenarchaeon have contributed to the understanding of novel DNA damage‐responsive regulation networks^30^, ^31^, ^32^, the archaeal cell division system^33^, ^34^, and cell cycle regulation^35^, ^36^. Versatile genetic tools have been developed for this model, including conventional and novel schemes of genetic manipulations^23^, ^37^, highly efficient expression systems^38^, ^39^, and CRISPR‐based mutagenesis and gene silencing^40^, ^41^, ^42^. We aimed to test a dual host–vector system for *Sa. islandicus* REY15A to further enrich its genetic toolbox. CRISPR escape mutations were designed on pRN1 and tested for their capability to overcome CRISPR restrictions to the plasmid, yielding highly efficient double‐plasmid vector systems for this model crenarchaeon.

## RESULTS AND DISCUSSION

### Spacer L2S56 in the CRISPR array of *Sa. islandicus* REY15A facilitates the I‐A immunity but fails to trigger immune responses from III‐B systems

**Figure 1:**
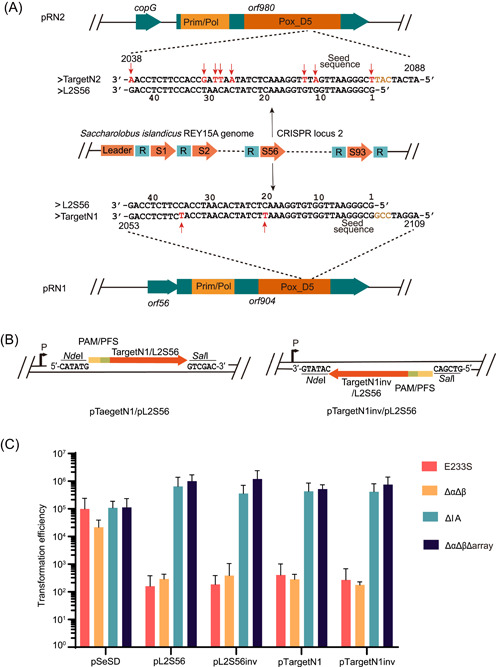
Restriction of pRN1‐derived plasmids by CRISPR–Cas systems in *Saccharolobus islandicus* REY15A. (A) Sequence alignment of Target N1/Target N2 and L2S56. L2S56, spacer 56 in CRISPR locus 2 of the host genome; Target N1, the pRN1 sequence matching the L2S56 spacer; Target N2, the pRN2 sequence showing similarity to the L2S56 spacer. Mismatches are highlighted in red and indicated with red arrows. The trinucleotides at the PAM position in Target N1 and non‐PAM motif position in Target N2 are highlighted in brown. (B) Schematic of the interference plasmids. The artificial L2S56 protospacer (L2S56) and Target N1 contain the 5′ flanking sequence 5′‐AGGATCCG‐3′ of the pRN1 target. (C) Testing CRISPR immunity using two sets of interference plasmids in REY15A. Four strains were employed for transformation, including the following: E233S, the wild‐type host; ΔIA, derived from E233S, lacking the *cas* gene module of I‐A CRISPR‐Cas; ΔαΔβ, derived from E233S lacking the Cmr‐α and Cmr‐β modules of *cas* genes; and ΔαΔβΔarray, derived from ΔαΔβ with both CRISPR arrays also deleted. PAM, protospacer adjacent motif; PFS, protospacer‐flanking sequence.

In our first attempts to construct shuttle vectors for *Sa. islandicus* REY15A, both pRN1 and pRN2 plasmids, the cryptic plasmids that coexist in the *Sa. islandicus* REN1H1^25^, ^43^, ^44^, were individually employed as an archaeal origin of replication in combination with the *Escherichia coli* cloning vector pGEM3z, and the resulting plasmid constructs were then tested by transformation of *Sa. islandicus* E233S, the genetic host derived from REY15A^45^. It has been reported that while pRN2‐derived plasmids scored a high transformation rate and yielded true transformants^23^, pRN1‐based vectors yielded only very few colonies from which plasmids were apparently absent^45^. Since CRISPR arrays often carry spacers matching various plasmids in *Sulfolobales* ^46^, ^47^, we suspected that the genome of *Sa. islandicus* REY15A might carry a spacer that matches a sequence in pRN1 but not in pRN2. After determining the complete genome sequence of *Sa. islandicus* REY15A^48^, it became clear that the host genome does carry a spacer homologous to a DNA segment in the pRN1 plasmid. It is the 56th spacer in the Locus 2 CRISPR array (L2S56), and the matching DNA segment (Target N1, Figure 1A) is within the coding sequence of *orf904*. Target N1 was 44 bp long, with two mismatches to the spacer positioned at 20 and 35 nt of the target sequence. Since the archaeal host carries one I‐A and two III‐B CRISPR‐Cas systems (Figure S1), we analyzed whether Target N1 could be recognized for destruction by these host CRISPR immune systems. Previous works have shown that a functional protospacer adjacent motif (PAM) at the 5′‐flanking position is required to elicit I‐A immunity in *Saccharolobus* ^49^, ^50^, ^51^. Target N1 is immediately downstream of the 5′‐CCG‐3′ trinucleotide (Figure 1A), which is one of the strong PAMs for I‐A systems. Thus, this DNA sequence is very likely to be targeted for destruction by the host I‐A CRISPR‐Cas. Furthermore, Target N1 is on the template strand of *orf904*, meaning that the mRNAs of the replicase gene are complementary to the crRNAs of L2S56. Since there are four mismatches between the upstream protospacer flanking sequence (PFS, 5′‐ATCCG‐3′) and the 5′‐GAAAG‐3′ repeat tag of crRNAs in *Sa. islandicus* REY15A^52^, upon transcription, the mRNAs of *orf904* contain a cognate target RNA sequence that could trigger the immune responses of the host III‐B Cmr systems (Figure S1), both of which are active in antiviral defense^53^, ^54^, ^55^, ^56^. On the other hand, L2S56 exhibits eight mismatches to the corresponding segment in pRN2, including a non‐PAM motif (5′‐CAT‐3′, Figure 1A), which would render the host CRISPR immunity ineffective against pRN2 in *Sa. islandicus* REY15A.

To test the above assumptions, target sequences were designed for L2S56 (ProtoL2S56) and Target N1, including the 5′‐CCG‐3′ PAM and 44 bp target sequences (Figure 1B). The resulting target modules were cloned to pSeSD in both orientations, yielding two types of plasmids, one with the same orientation as the promoter (i.e., pTarget N1 and pL2S56), whereas the other was in the reverse orientation (i.e., pTarget N1inv and pL2S56inv) (Figure 1A,B). Theoretically, both types of target plasmids are recognized by the I‐A system, but only the plasmids carrying the inverted target sequence can produce cognate target RNAs that activate the immune responses of III‐B CRISPR systems in this archaeon^53^, ^57^, ^58^.

**Figure 2:**
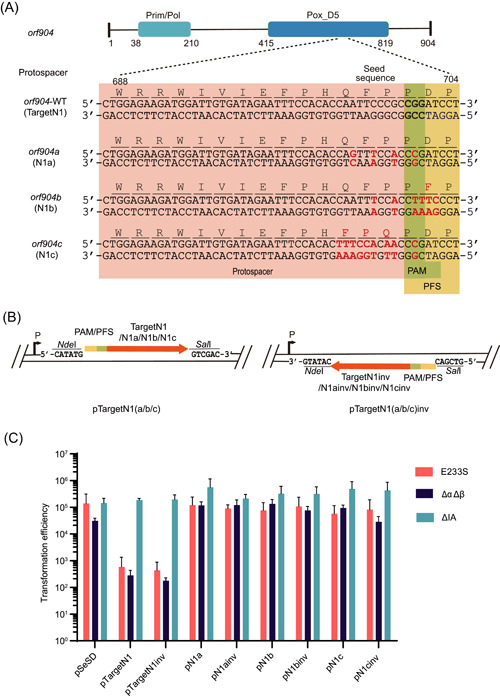
Rational design of Target N1 mutations and testing their capability to evade CRISPR immunization in *Sa*. *islandicus* REY15A. (A) The wild‐type target sequence region and three mutated ones (N1a, N1b, and N1c) are highlighted in the pink region, and the flanking sequences are in the green (protospacer adjacent motif) and yellow (protospacer‐flanking sequence) regions. Mutations in both DNA and protein sequences are highlighted in red. (B) The design of protospacer modules in both orientations for the three derivative DNA segments based on pRN1 N1a, N1b, and N1c. (C) Transformation efficiencies of the corresponding protospacer/target plasmids. Three hosts were employed for transformation, including E233S, ΔIA, and ΔαΔβ.

These plasmids were then employed to test their capability to induce immune responses in the wild‐type E233S strain and in three mutants carrying deletions in distinctive sets of CRISPR elements (Table S1), including the following: (a) ΔαΔβ in which both III‐B modules were removed, thus carrying only the I‐A CRISPR system^41^; (b) ΔαΔβΔarray, which was derived from ΔαΔβ by deleting both CRISPR arrays^56^; the immune system in this mutant should be inactive unless crRNAs can be provided from a CRISPR plasmid; and (c) ΔIA lacking *cas* genes of the I‐A interference module^59^. Transformation of the four archaeal hosts with pL2S56 yielded the following results: (a) while the reference plasmid pSeSD scored a very similar rate of transformation (0.2−1 × 10^5^ colonies/μg) in all the four strains, electroporation of pL2S56 or pL2S56inv into transformation of E233S and ΔαΔβ showed  >100‐fold reduced level of transformation (ca. 2 × 10^2^ colonies/μg), indicative of the I‐A immune responses with these interference plasmids; and (b) the immunity was abolished both in ΔIA and in ΔαΔβΔarray since each interference plasmid scored a transformation rate of ca. 10^6^ colonies/μg, which is even higher than that of the reference plasmid pSeSD (Figure 1C). The results showed that plasmid‐induced CRISPR immunity is dependent on the CRISPR arrays and the I‐A CRISPR module but III‐B CRISPR‐independent. Very similar results were obtained with pTarget N1 and pTarget N1inv, indicating that the two mismatches between the pRN1 target and L2S56 did not influence the induction of the I‐A immune responses (Figure 1C). Neither pL2S56inv nor pTarget N1inv induced III‐B responses in this archaeon (Figure 1C). In a previous study, it was shown that a relatively higher level of crRNA/target RNAs is required for triggering immunity of III‐B CRISPR‐Cas compared with the I‐A systems^56^. These data suggest that L2S56 crRNAs were expressed to a level that could be sufficient to elicit the I‐A immunity but insufficient to trigger the III‐B immunity for plasmid elimination in this archaeon.

### Identification of functional derivatives of pRN1 *orf904* that evade L2S56‐driven CRISPR immunity in the archaeal host

To identify a functional target N1 derivative that evades the host CRISPR immunity, three DNA segments were designed based on the pRN1 target (Figure 2A). The first mutant (N1a) possessed 5′‐CGG‐3′ motif at the PAM position and carried three additional mismatches at the −2, −5, and −7 positions in the seed sequence region. The second mutant (N1b) carried six nucleotide sequence changes, including two mismatches at the −2 and −5 positions in the seed sequence region and four changes at the PFS motif, yielding the 5′‐GAAAG‐3′ motif at the target site. The last change made a noncognate target RNA upon transcription. The third mutant (N1c) had nine mutations. Noticeably, while the designed mutations in N1a were synonymous, N1b and N1c had missense mutations: Those in N1b led to the change of one amino acid and those in N1c led to three substitutions (Figure 2A).

These DNA fragments were then cloned to pSeSD in both orientations (Figure 2B), and the resulting plasmids were tested for their ability to induce CRISPR immunity in this archaeon using the interference plasmid assay. Specifically, three hosts were employed for transformation, including the following: E233S, the wild‐type strain; ΔαΔβ, the I‐A CRISPR‐active strain, and ΔIA, the III‐B CRISPR‐active strain (Table S1). The results showed that while Target N1 induced immune responses from the I‐A CRISPR system, none of the three mutated targets (i.e., N1a, N1b, and N1c) were targeted by the CRISPR system in the archaeal host (Figure 2C). Therefore, all the designed protospacer mutations successfully evaded CRISPR immunity in *Sa. islandicus* REY15A, which is in agreement with the results obtained with the interference plasmid assays shown in Figure 1C.

**Figure 3:**
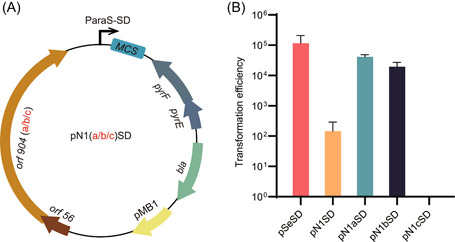
Functionality of the mutated *orf904* genes carrying a mutated pRN1 protospacer. (A) Construction of vectors with each mutated *orf904* gene. The *Saccharolobus* selection marker *Escherichia coli* replicon (pMB1) and selection marker (*bla*) were derived from pSeSD. The *Saccharolobus* replicon was obtained from pRN1. Ligation of these two parts yielded pN1SD. Nontarget DNA fragments of pRN1 N1a/b/c were designed (Figure 2A) and employed to replace the wild‐type pRN1 replicon, giving pN1aSD, pN1bSD, and pN1cSD. (B) Transformation rates of the constructed pRN1‐derived vectors. All the constructed *Saccharolobus* vectors were introduced into the archaeal cells by electroporation. Colonies of transformants were obtained on sucrose, casamino acids, and vitamin plates and enumerated. Transformation efficiencies are shown as numbers of transformants/µg DNA.

These mutated DNA segments were then individually incorporated into *orf904* by inverse PCR with pN1SD as the template. Gibson assembly of the resulting linear PCR fragments yielded pN1aSD, pN1bSD, and pN1cSD, respectively. The synonymous mutations, as well as the missense mutations carried by the plasmids, are shown in Figures 2A and S2. These plasmids were introduced into *Sa. islandicus* by electroporation to test the functionality of the pRN1 derivatives. As shown in Figure 3, only a few colonies of transformants were obtained with pN1SD, indicating that the new wild‐type pRN1‐based shuttle vector was also restricted in *Sa. islandicus* RAY15A, as seen for pREF11 in the same host^45^. We also found that pN1cSD did not yield any colonies in the transformation, suggesting that the carried missense mutations could have inactivated the replication protein. This is also in agreement with the location of the target site in the conserved Pox_D5 domain of the pRN1 primase/polymerase^60^, ^61^, ^62^. To this end, two mutated replicase genes (*orf904a* and *orf904b*) of pRN1 were found to be functional in wild‐type *Sa. islandicus* REY15A.

### Testing the dual vector system with *Sa. islandicus pyrEF argD* double mutants

**Figure 4:**
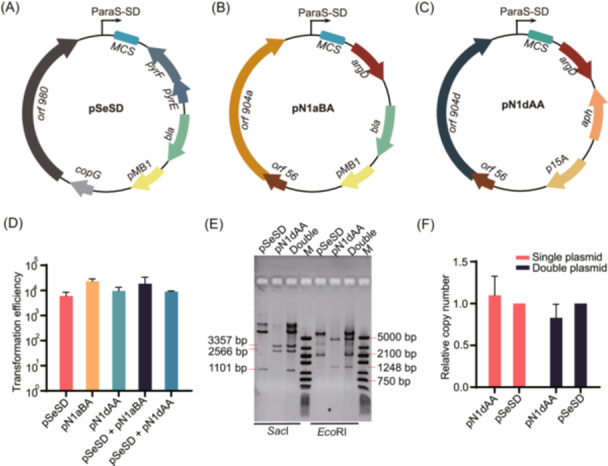
Compatibility of the pRN2‐ and pRN1‐derived vectors in *Sa*. *islandicus*. (A–C) Maps of different plasmid vectors for *Sa. islandicus*. pSeSD, a pRN2‐based vector with the *pyrEF* selection marker; pN1aBA, a pRN1 N1a‐based vector with the *argD* gene as the selection marker; pN1dAA, pN1aBA‐derived vector in which the *Escherichia coli* pMB1 replicon was replaced with the p15A origin, along with the change of selection marker from *bla* (ampicillin resistance) to *aph* (kanamycin resistance). (D) Transformation efficiencies with single plasmids or double plasmids. These vectors were introduced into the archaeal cells by electroporation. Colonies of transformants were obtained on sucrose, casamino acids, and vitamin plates and enumerated. Transformation efficiencies are shown as numbers of transformants/µg DNA. (E, F) Relative copy number of pSeSD (pRN2‐derived) and pN1dAA (pRN1‐derived) vectors. Gray values of DNA bands were quantified using ImageJ software and normalized by the length of each fragment (kb) (E). Relative copy numbers were then calculated, with the copy number of pSeSD set to 1.0 (F). Single plasmid, either pSeSD or pN1dAA; double plasmids, pSeSD + pN1dAA; M, DNA marker ladder.

Previously, an *Sa. islandicus pyrEF argD* double mutant (E233SA; Table S1) was constructed and employed in testing microhomology‐mediated high‐throughput gene inactivation in comparison with *Sa. islandicus* M.16.4^61^. The genetic host can be selected with two genetic markers, one based on the complementation of the *pyrEF* gene and the other based on *argD* complementation. Thus, it represents a suitable host for testing a dual plasmid system. A series of vectors was constructed to yield a suitable vector for testing the dual plasmid system (Figure S3). The first step was to construct a pRN1‐derived shuttle vector with *argD* as the selection. This was done by amplifying an *argD* gene from *Saccarolobus solfataricus*, with which the *pyrEF* marker in pN1aSD was replaced, producing pN1aBA. To facilitate cloning with the vector, *Nde*I in *orf904* was removed by reverse PCR and subsequent Gibson assembly, leading to pN1dBA. However, pSeSD and pN1dBA still shared two elements: the pMB1 origin and the *bla* marker. To eliminate this redundancy, a different bacterial origin of replication and a kanamycin‐resistant marker were amplified by PCR from p15AIE and pET30a, respectively. Assembling them with the *Saccharolobus* replicon of pN1dBA yielded pN1dAA, which only shared a promoter and a multiple cloning site with pSeSD (Figures 4A–C and S3).

These vectors were then used to transform E233SA, either alone or in combination. As shown in Figure 4D, the transformation of *Sa. islandicus* cells, either with each plasmid individually or with two plasmids simultaneously, yielded very similar efficiencies. These results indicate that these plasmid vectors are fully compatible with each other in the same cell, consistent with the concurrence of their wild‐type plasmids in the original *Sa. islandicus* strain^25^.

To test whether the two plasmid vectors could be maintained at similar levels in host cells, strains carrying pSeSD, pN1dAA, or both were grown in selective media to a late growth phase. The cell mass was then harvested for plasmid extraction. The resulting plasmid DNA was then analyzed by restriction digestion and agarose gel electrophoresis. As shown in Figure 4E, restriction fragments of the predicted sizes appeared for each plasmid. Their relative ratios in the cell were determined by calculating the gray value of the enzymatic digestion products using the values of pSeSD as the reference (100%). This yielded the result that the relative level of the two vectors in the single plasmid system was very similar to the cells carrying both plasmids (Figure 4F). To assess plasmid maintenance in *Sa. islandicus*, these cultures were grown for 16 consecutive transfers (a dilution of 20–25‐fold was made in each transfer when the OD_600_ values of these cultures reached 0.8–1.0). Plasmid DNA was then prepared from the cell mass collected from the last batch of cultures, and the relative yields of plasmid were very similar to those prepared from the first batch of cultures, as shown in Figure 4E. These results show that both vectors are stably maintained in *Sa. islandicus* under the selection.

*Sulfolobales* organisms are widespread in hot springs around the world^62^. These organisms often coexist with extrachromosomal genetic elements, including viruses and cryptic and conjugative plasmids^63^. In the CRISPR target database^64^, reference sequences of plasmids and viruses available in the public GenBank databases are included, allowing CRISPR target sequences to be determined by searching the databases with sequences of CRISPR arrays. We conducted a search with the CRISPR arrays of *Sa. islandicu*s REY15A and found that several additional plasmids contain targets of the CRISPR spacers in this archaeon, including that: (a) one conjugative plasmid, pANR4, carries a protospacer that exhibits a perfect match to Spacer 28 in Locus 1 (L1S28) of the CRISPR arrays; (b) three other conjugative plasmids (pING1, pKEF9, and pMGB1) contain protospacers that respectively show one mismatch to L1S28, L1S29, and L2S44 (Table S2), and these elements are also likely to be targeted in this archaeon. Investigation of pKEF9 conjugation in *Sa. islandicu*s revealed that the pKEF9‐targeting spacer was eliminated in conjugated cells via spacer deletion during conjugation^65^. Moreover, four other plasmids (cryptic plasmid pHEN7, conjugative plasmids pARN3, pARN4, and pAH1) exhibited five mismatches to several spacers (e.g., L2S1 showed five mismatches to pARN3 and pARN4; Table S2). Since III‐B systems can tolerate mismatches between crRNAs and their targets^41^, ^53^, ^57^, ^58^, ^66^, it would be interesting to investigate whether some of these spacers could be targeted by the host CRISPR immune systems and, if so, whether they could be silenced differentially by the I‐A and III‐B CRISPR systems in this archaeon.

In conclusion, we demonstrate that pRN1 will be targeted by the I‐A CRISPR system of *Sa. islandicus* REY15A, since it carries a DNA segment in *orf904* coding for the plasmid replicase, and the design of a pRN1 mutant escaping host immunity by changing the CRISPR target sequence to a nontarget one yields a fully functional plasmid backbone for vector construction.

## MATERIALS AND METHODS

### Strains and plasmids used in this study and cultivation

*Sa. islandicus* strains and plasmids used in this study are listed in Table S1. These archaea were grown in sucrose, casamino acids, and vitamin medium or in d‐arabinose, casamino acids, and vitamin medium^23^. If required, both uracil and agmatine were supplemented to 20 μg/ml, for cultivation of mutant strains. Incubation was at 75°C with shaking at 200 rpm. Electroporation transformation of *Saccharolobus* cells was conducted with ca. 600 ng plasmid DNA, following the procedure previously described^23^, ^67^.

### Enzymes and reagents employed in this study

All oligonucleotides employed in this study (Table S3) were synthesized in Tsingke (Qingdao), and restriction enzymes were purchased from Thermo Fisher Scientific. Amplified PCR products were purified using a Cycle‐Pure kit (Omega). The designed plasmids were recovered by transformation of *E. coli*, with designed mutated sequences confirmed by DNA sequencing in Tsingke (Qingdao). Omega plasmid extraction kits for plasmid extraction were purchased from Omega.

### Construction of target plasmids and plasmid interference assay

Five DNA fragments were designed carrying a wild‐type or mutated target, that is, the protospacer of the 56th spacer in locus 2 in the CRISPR array of the genetic host E233S (Proto‐L2S56), its matching sequence in pRN1 (TargetN1) as well as three mutated targets (N1a, N1b, and N1c) that contain mutations in the seed sequence region and/or mismatches in protospacer‐adjacent motifs for the I‐A CRISPR‐Cas^49^, ^51^ and protospacer‐flanking sequences for the III‐B immune systems^41^, ^53^, ^57^, ^58^ in *Sa. Islandicus* REY15A. Each DNA fragment was obtained by the alignment of two overlapping oligos (Table S3) and subsequent polymerase extension. These DNA fragments were digested with LguI enzyme and cloned into pSeSD^38^, yielding plasmid constructs pL2S56, pTargetN1, pN1a, pN1b, and pN1c, which carry the wild‐type L2S56 protospacer, target‐N1, or each of the mutated targets (Table S1).

Interference plasmid assays were conducted as previously described^49^, ^53^, and a great reduction in transformation rate with a test plasmid compared with the corresponding reference (>10‐fold) is indicative of immune responses elicited by a target sequence on the plasmid^28^.

### Construction of pRN1‐based vectors

To construct pRN1‐based *Saccharolobus–E. coli* shuttle vectors, a DNA fragment containing an *E. coli* replicon and the *pyrEF* selection marker (4196 bp) was amplified from pSeSD^38^ by PCR with primer pairs pSD‐F/pSD‐R, and the predicted minimal replicon of pRN1^68^, including the *orf56* and *orf904* genes, was amplified from pREF11^45^ by PCR using the primer pair of pREF‐orf904‐F/pREF‐orf904‐R. Subsequently, the two PCR fragments were circularized by the Gibson assembly^69^ based on the *E. coli* RedET system^70^ to yield pN1SD (Figure S1). The plasmid was then recovered by transformation of *E. coli* and confirmed by restriction analysis.

To generate pN1SD derivatives carrying mutated targets in *orf904* (see the Results and Discussion section), primers N1a‐F/N1a‐R, N1b‐F/N1b‐R, and N1c‐F/N1c‐R were designed and employed in inverse PCR reactions using Phanta Max Super‐Fidelity DNA Polymerase (Vazyme Biotech) with pN1SD as the template. The resulting linear fragments containing N1a, N1b, or N1c DNA segment were self‐circularized using the Gibson assembly to yield pN1aSD, pN1bSD, and pN1cSD, respectively (Table S1 and Figure S1). These plasmids were recovered by transformation of *E. coli* and their mutated targets were confirmed by DNA sequencing.

To construct pRN1 vectors with the *argD* selection, the marker gene was amplified by PCR from *S. solfataricus* P2^71^ using primer pairs P2‐argD‐F/P2‐argD‐R. In addition, a DNA fragment of pN1aSD lacking *pyrEF* was generated by inverse PCR using primer pairs pN1aSD‐F/pN1aSD‐R. The *argD* marker gene was fused together with the pN1aSD linearized fragment via Gibson assembly, giving the pN1aBA plasmid with the *bla* (beta‐lactamase) marker in *E. coli* and the *argD* marker in *Sa. islandicus*. In addition, the occurrence of a *Nde*I restriction site in *orf904* prevents the utilization of *Nde*I in the multiple cloning sites region (MCS) for cloning. To remove that restriction site, primers N1‐mNL‐F and N1‐mNL‐R were designed and employed for *Nde*I site mutagenesis with SOE‐PCR to yield pN1dBA (Figure S2).

To construct novel pRN1‐based vectors, an *E. coli* replicon and selection marker different from those present on pSeSD should be used. To do that, DNA fragments containing the *E. coli* p15A origin were amplified by PCR from p15AIE using k15aori‐F/15aori‐N1‐R primers, whereas the *aph* gene encoding an aminoglycoside phosphotransferase was amplified from pET30a using ak‐F/k15aori‐R primers. The third fragment carrying the *Saccharolobus* origin of replication and the *argD* marker was obtained by PCR from pN1dBA using 15aori‐N1‐F and ak‐R primers. The resulting three fragments were recombined using the Gibson assembly reaction to yield the pN1dAA plasmid (Figure S1).

### Determination of the relative content of pRN1‐ and pRN2‐derived plasmids

Relative plasmid content was determined by gel quantification of DNA bands after agarose gel electrophoresis. *Sa. islandicus* E233SA strains containing pSeSD, pN1dAA, and both of the above plasmids, respectively, were cultured to the OD_600_ of 0.8–1.0. Plasmid DNAs were extracted from cell mass using an Omega plasmid extraction kit. Equal volumes of plasmid preparations were respectively digested with *Sac*I or *Eco*RI at 37°C for 1 h. ImageJ software^72^ was used to quantify the gray value ratio of digestion product bands. The resulting values were normalized by the length of DNA fragments, yielding relative gray values/kb DNA fragment for each plasmid, which were used to calculate the relative copy number of pN1dAA and pSeSD plasmid in the cell.

## AUTHOR CONTRIBUTIONS

**Pengpeng Zhao**: Data curation (equal); formal analysis (equal); investigation (equal); methodology (equal); validation (equal); visualization (equal); writing—original draft (supporting); writing—review and editing (supporting). **Xiaonan Bi**: Data curation (equal); formal analysis (equal); investigation (equal); methodology (equal); validation (equal); visualization (supporting); writing—original draft (supporting). **Xiaoning Wang**: Data curation (equal); formal analysis (equal); investigation (equal); methodology (equal); validation (supporting); visualization (supporting). **Xu Feng**: Formal analysis (equal); funding acquisition (supporting); methodology (equal); validation (equal); visualization (supporting); writing—original draft (supporting); writing—review and editing (supporting). **Yulong Shen**: Formal analysis (equal); methodology (supporting); resources (supporting); validation (equal); visualization (supporting); writing—review and editing (supporting). **Guanhua Yuan**: Data curation (equal); formal analysis (equal); investigation (equal); methodology (equal); supervision (equal); validation (equal); visualization (supporting); writing—original draft (equal); writing—review and editing (supporting). **Qunxin She**: Conceptualization (lead); formal analysis (equal); funding acquisition (lead); methodology (equal); project administration (lead); resources (equal); supervision (lead); validation (equal); visualization (equal); writing—original draft (equal); writing—review and editing (lead).

## ETHICS STATEMENT

No animal or human experiments were involved in this study.

## CONFLICT OF INTERESTS

The authors declare no conflict of interests.

## Supporting information

Supporting information.

## Data Availability

All relevant data have been reported in the submitted article, either in the manuscript or in the supplementary data file.
