# Supplementary material for: Rational design of unrestricted pRN1 derivatives and their application in the construction of a dual plasmid vector system for Saccharolobus islandicus
Source: mLife. 2024 Mar 20;3(1):119–28. doi: 10.1002/mlf2.12107 (PMC11139203; doi:10.1002/mlf2.12107)
Supplement: Supplementary file 1 — Supporting information. [file MLF2-3-119-s001.pdf]

Supplementary figures and tables to

Rationale design of unrestricted pRN1 derivatives and their application in construction of a dual plasmid vector system for *Saccharolobus islandicus*

Pengpeng Zhao[1], Xiaonan Bi[1], Xiaoning Wang, Xu Feng, Yulong Shen, Guanhua Yuan*, Qunxin She*

CRISPR and Archaea Biology Research Center, State Key Laboratory of Microbial Technology and Microbial Technology Institute, Shandong University, Qingdao 266237, China

* Authors for correspondence: email (shequnxin@sdu.edu.cn; yuangh@sdu.edu.cn)

[1] Co-first authors

This file contains 3 supplementary figures and 3 supplementary tables.

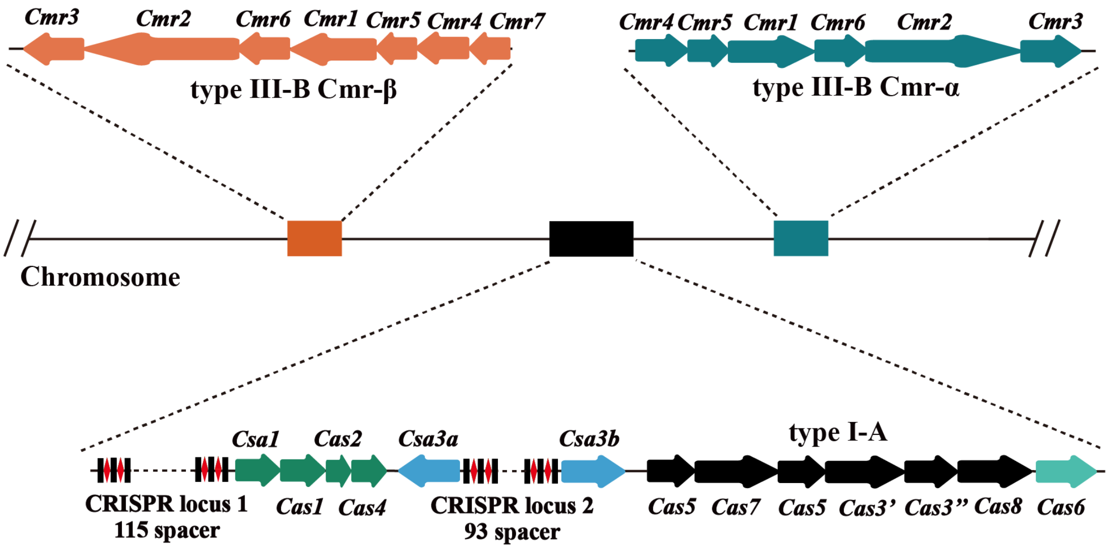

Figure S1 CRISPR-Cas systems encoded in *Sa islandicus* REY15A. Type I-A systems mediates the protospacer-adjacent motif-dependent DNA interference (1); Type III-B CRISPR systems (Cmr-α and Cmr-β) mediate the transcription-dependent interference of protospacer (2). The immunity is activated by cognate target RNAs showing mismatches to the 5'-repeat tag sequence of crRNA (TGAAAG) (3, 4, 5), yielding invader clearance or cell dormancy or cell death (6, 7, 8). CRISPR locus 1 carries 115 spacers whereas locus 2 has 93 spacers. In addition, the host also carries a *cas* gene locus of adaptation (Csa1, Cas1, Cas2 and Cas4) responsible for spacer acquisition and two CRISPR-associated transcriptional regulators (Csa3a and Csa3b).

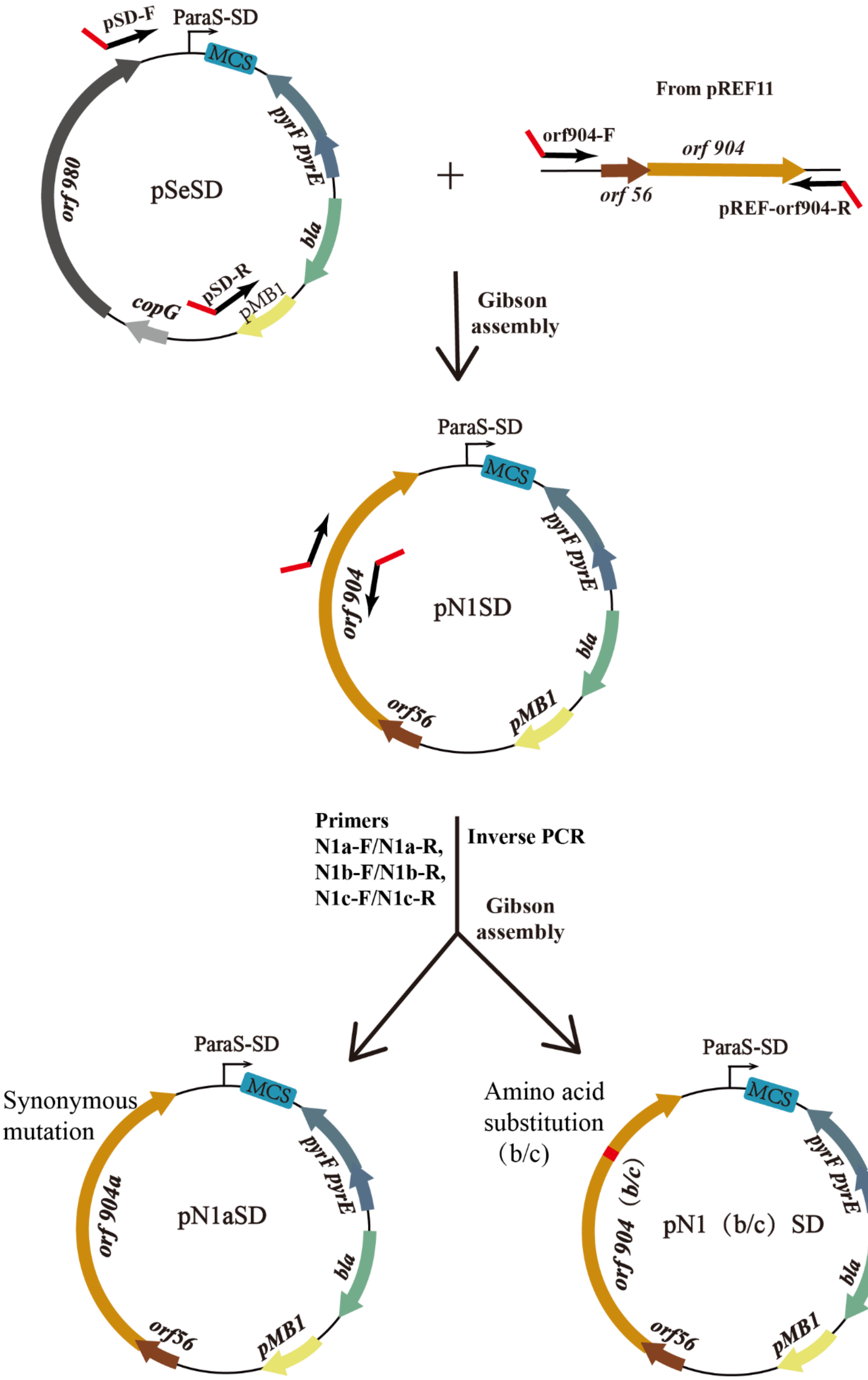

Figure S2 Construction of the pRN1-based *Saccharolobus-E. coli* shuttle vectors.

A DNA fragment containing an *E. coli* replicon and the *pyrEF* selection marker (4196bp) from pSeSD plasmid and the predicted minimal replicon (including the *orf56* and *orf904* genes) of pRN1 from pREF11 were amplified with the primer pairs pSD-F/pSD-R and pREF-orf904-F/pREF-orf904-R, respectively. Subsequently, the two PCR fragments were circularized by Gibson assembly to yield pN1SD. To generate pN1SD derivatives carrying mutated protospacers in *orf904*, overlapping primers N1a-F/N1a-R, N1b-F/N1b-R and N1c-F/N1c-R containing the desired mutation were used for reverse PCR by using pN1SD as the template to introduce the *orf904a*, *orf904b* and *orf904c* into complementing plasmid pN1aSD, pN1bSD and pN1cSD, respectively.

pyrE: Orotate phosphoribosyltransferase; *pyrF*: orotidine-5′-monophosphate decarboxylase; *bla*: beta-lactamase.

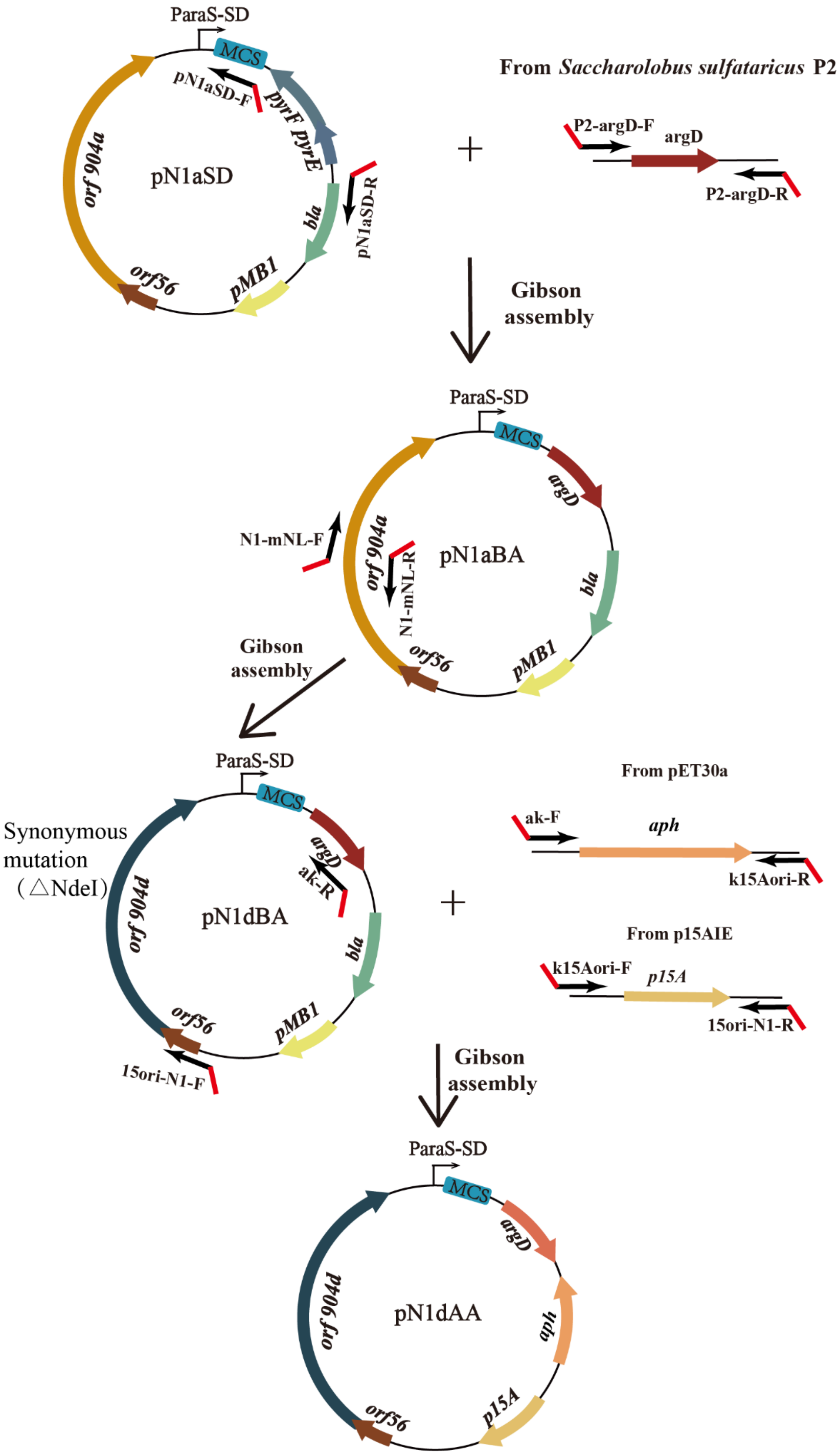

Figure S3 Construction of the pRN1-based *Saccharolobus-E. coli* shuttle vectors with an *E. coli*

replicon and selection marker different from those on pSeSD.

The *argD* selection marker gene from *Sa. solfataricus* P2 using primer pairs P2-argD-F/P2-argD-R was fused via Gibson assembly into the linearized pN1aSD lacking *pyrEF*, which was prepared by inverse PCR with the primer set pN1aSD-F/pN1aSD-R, to yield the pN1aBA plasmid. To remove the NdeI restriction site in *orf904*, primers N1-mNL-F and N1-mNL-R were designed and employed for NdeI site mutagenesis with SOE-PCR to yield pN1dBA. The *aph* selection marker gene amplified with k15aori-F/15aori-N1-R from pET30a and the p15aori amplified with ak-F/k15aori-R from p15AIE were combined with the PCR linearized pN1dBA plasmid using 15aori-N1-F/ak-R for Gibson assembly to form the final plasmid pN1dAA.

aph: an aminoglycoside phosphotransferase gene; *argD*: an acetylornithine aminotransferase gene.

Supplementary Tables

Table S1 *S. islandicus* strains and plasmids used in this work

Strain or plasmid*	Description	Reference
Strains-*S. islandicus*		
**E233S**	**Δ*pyrEF*, Δ*lacS***	**(9)**
ΔIA-E233S	Derived from S. islandicus E233S, carrying deleting of *cas* genes of the I-A interference module	(10)
ΔαΔβ	Derived from *S. islandicus* E233S, carrying deleting of both III-B modules	(4)
ΔαΔβΔarray	Derived from *S. islandicus* ΔαΔβ by deletion of both CRISPR arrays	(11)
**E233SA**	**Δ*pyrEF*, Δ*lacS*, Δ*argD***	
Plasmids		
**pSeSD**	**A *Sulfolobus-E. coli* shuttle vector derived from pRN2**	**(12)**
pREF11	An unstable *Sulfolobus*-*E. coli* shuttle vector derived from pRN1	(13)
pL2S56	pSeSD carried a L2S56-protospacer	This work
pL2S56inv	pSeSD carried an inverted L2S56-protospacer	This work
pTargetN1	pSeSD carried a pRN1-protospacer	This work
pTargetN1inv	pSeSD carried an inverted pRN1-protospace	This work
pN1a/b/c	pSeSD carried the mutant pRN1-protospacer	This work
pN1a/b/c-inv	pSeSD carried h the inverted mutant pRN1-protospacer	This work
pN1SD	A pRN1-based *Sulfolobus*-*E. coli* shuttle plasmid, consisting of an orf56-orf904 fragment of pRN1 fused to a 0-4196 bp fragment of pSeSD, carrying the *pyrEF* gene	This work
pN1a/b/cSD	Mutation of the *orf904* sequence in the pN1SD	This work
pN1aSA	Replace *pyrEF* gene with *argD* gene in pN1aSD	This work
pN1dSA	Mutation of the NdeI site of *orf904* in pN1aBA	This work
**pN1dKA**	**Replace *Amp*^{r} gene with *Kan*^{r} gene, and *pMB1* with p15A-*ori* in pN1dBA**	**This work**

*Genetic hosts and plasmid vectors for *Sa islandicus* REY15A are highlighted in bald face.

Table S1 Oligonucleotides and primers used in this work

Name	Oligonucleotides (5′–3′)
pSD-F	CGTGATAATATTTGTATAGTAAGCATGCATGTTAAACAAG
pSD-R	GTGAAAAGCTAATCTCGAGGCTTCCTCGCTCACTGAC
pREF-orf904-F	GTCAGTGAGCGAGGAAGCCTCGAGATTAGCTTTTCAC
pREF-orf904-R	CTTGTTTAACATGCATGCTTACTATACAAATATTATCACG
P2-argD-F	TCTTTTTTTTCCCGGGAAGATAAAATATTGTTGCG
P2-argD-R	CACTATAGGGCGAATTCGGGGTACTTTCTTACTGC
pN1aSD-F	GCAGTAAGAAAGTACCCCGAATTCGCCCTATAGTG
pN1aSD-R	CGCAACAATATTTTATCTTCCCGGGAAAAAAAAGA
N1a-F	TTTCCACCTTTCCCTAACTTTTTCGATAAGACATTC
N1a-R	AAAGTTAGGGAAAGGTGGAAACTGGTGTGGAAATTC
N1b-F	CCAGTTTCCACCCGATCCTAACTTTTTCG
N1b-R	TCGGGTGGAAACTGGTGTGGAAATTCTATC
N1c-F	CCACACTTTCCACAACCCGATCCTAACTTTTTCGATAAG
N1c-R	GGGTTGTGGAAAGTGTGGAAATTCTATCACAAT
N1-mNL-F	CATCTGTCACTGGATTGATGAGGAACACGTGCGGTTTGATTG
N1-mNL-R	CGTGTTCCTCATCAATCCAGTGACAGATGTTTCTCTTCTCGC
15aori-N1-F	AGCCTTTTTTCTCGAGATTAGCTTTTCACAC
15aori-N1-R	GCTAATCTCGAGAAAAAAGGCTGCACCGGTG
ak-F	AGAAAGTACCCCAGGTGGCACTTTTCGGGGA
ak-R	GTGCCACCTGGGGTACTTTCTTACTGCTTTG
k15aori-F	AAGGATCTTCGGCGGTTTGCGTATTGGCTA
k15aori-R	GCAAACCGCCGAAGATCCTTTGATCTTTTCTACG
N1-argD-F	TTTTCCCGGGAAGATAAAATATTGTTGCGACTGAG
N1-argD-R	ATTTTATCTTCCCGGGAAAAAAAAGATTTTGC
Proto-N1-F	TATTCTGGAGAAGATGGATTGTGATAGAATTTCCACACCAATT CCCGCCGGATCCT
Proto-N1-R	TCGAAGGATCCGGCGGGAATTGGTGTGGAAATTCTATCACAA TCCATCTTCTCCAGAA
ProtoL2-S56-F	TTCTGGAGAAGGTGGATTGTGATAGAGTTTCCACACCAATTC CCGCCGGATCCT
ProtoL2-S56-R	AGGATCCGGCGGGAATTGGTGTGGAAACTCTATCACAATCCA CCTTCTCCAGAA
Proto-N1a-F	TATTCTGGAGAAGATGGATTGTGATAGAATTTCCACACCAATT TCCACCTTTCCCT
Proto-N1a-R	TCGAAGGGAAAGGTGGAAATTGGTGTGGAAATTCTATCACAA TCCATCTTCTCCAGAA
Proto-N1b-F	TATTCTGGAGAAGATGGATTGTGATAGAATTTCCACACCAGTT TCCACCCGTCCCT
Proto-N1b-R	TCGAAGGGACGGGTGGAAACTGGTGTGGAAATTCTATCACAA

	TCCATCTTCTCCAGAA
Proto-N1c-F	TATTCTGGAGAAGATGGATTGTGATAGAATTTCCACACTTTCC ACAACCCGTCCCT
Proto-N1c-R	TCGAAGGGACGGGTTGTGGAAAGTGTGGAAATTCTATCACAA TCCATCTTCTCCAGAA

Table S3 Plasmid-matching spacers in the genome of *S. islandicus* REY15A

CRISPR arrays	Spacers	Target plasmids (number of mismatched bases)
**CRISPR locus 1 （115 spacer）**	Spacer 1	pING1（8）、pKEF9（5）、pHVE14（8）、pNOB8（10）、pSOG2（8）
	Spacer 12	pB12E5（12）
	Spacer 23	pARN4（7）
	Spacer 25	pRN1（8）、pING1（6）
	Spacer 28	pHVE14（0）、pMGB1（1）
	Spacer 29	pING1（1）、pHVE14（10）、pSOG1（7）、pSOG2（7）、pAH1（5）、pLD8501（10）
	Spacer 30	pAH1（10）
	Spacer 34	pYN01（5）
**CRISPR locus 2 （93 spacer）**	Spacer 1	pARN3（5）、pARN4（5）、pMGB1（7）
	Spacer 44	pKEF9（1）
	Spacer 48	pKEF9（6）、pING1（9）
	Spacer 56	pRN1（2）、pHEN7（5）、pRN2（8）

References

1. Gudbergsdottir S, Deng L, Chen ZJ, Jensen JVK, Jensen LR, She QX, et al. Dynamic properties of the Sulfolobus CRISPR/Cas and CRISPR/Cmr systems when challenged with vector-borne viral and plasmid genes and protospacers. Molecular Microbiology. 2011;79:35-49.
2. Deng L, Garrett RA, Shah SA, Peng X, She Q. A novel interference mechanism by a type IIIB CRISPR-Cmr module in Sulfolobus. Mol Microbiol. 2013;87:1088-1099.
3. Sofos N, Feng M, Stella S, Pape T, Fuglsang A, Lin J, et al. Structures of the Cmr-beta complex reveal the regulation of the immunity mechanism of type III-B CRISPR-Cas. Mol Cell. 2020;79:741-757 e747.
4. Peng W, Feng M, Feng X, Liang YX, She Q. An archaeal CRISPR type III-B system exhibiting distinctive RNA targeting features and mediating dual RNA and DNA interference. Nucleic Acids Res. 2015;43:406-417.
5. Han W, Li Y, Deng L, Feng M, Peng W, Hallstrøm S, et al. A type III-B CRISPR-Cas effector complex mediating massive target DNA destruction. Nucleic Acids Research. 2017;45:1983-1993.
6. Rostol JT, Marraffini LA. Non-specific degradation of transcripts promotes plasmid clearance during type III-A CRISPR-Cas immunity. Nat Microbiol. 2019;4:656-662.
7. Zhang Y, Lin J, Tian X, Wang Y, Zhao R, Wu C, et al. Inactivation of Target RNA Cleavage of a III-B CRISPR-Cas System Induces Robust Autoimmunity in Saccharolobus islandicus. International Journal of Molecular Sciences. 2022;23:8515.
8. Yu Z, Xu J, Zhang Y, She Q. The influence of the copy number of invader on the fate of bacterial host cells in the antiviral defense by CRISPR-Cas10 DNases. Engineering Microbiology. 2023;3:100102.
9. Deng L, Zhu H, Chen Z, Liang YX, She Q. Unmarked gene deletion and host-vector system

for the hyperthermophilic crenarchaeon *Sulfolobus islandicus*. Extremophiles. 2009;13:735-746.
10. Peng W, Li H, Hallstrom S, Peng N, Liang YX, She Q. Genetic determinants of PAM-dependent DNA targeting and pre-crRNA processing in *Sulfolobus islandicus*. RNA Biol. 2013;10:738-748.
11. Zhang Y, Lin J, Tian X, Wang Y, Zhao R, Wu C, et al. Inactivation of target RNA cleavage of a III-B CRISPR-Cas system induces robust autoimmunity in *Saccharolobus islandicus*. Int J Mol Sci. 2022;23.
12. Peng N, Deng L, Mei Y, Jiang D, Hu Y, Awayez M, et al. A synthetic arabinose-inducible promoter confers high levels of recombinant protein expression in hyperthermophilic archaeon *Sulfolobus islandicus*. Appl Environ Microbiol. 2012;78:5630-5637.
13. She Q, Deng L, Zhu H, Chen Z, Dreibrøl M, Awayez M, et al. Host-vector systems for hyperthermophilic archaeon *Sulfolobus*. In: Liu SJ, Drake HL, editors. Microbes and the Environment: Perspective and Challenges. Beijing: Beijing: Science Press; 2008. p. 151–156.
